# Update on the risks of complications after knee arthroscopy

**DOI:** 10.1186/s12891-018-2102-y

**Published:** 2018-06-01

**Authors:** Katarina Friberger Pajalic, Aleksandra Turkiewicz, Martin Englund

**Affiliations:** 1 Department of Clinical Sciences Lund, Orthopaedics, Clinical Epidemiology Unit, Faculty of Medicine, Lund University, Remissgatan 4, 221 85 Lund, Sweden; 2 Clinical Epidemiology Research & Training Unit, Boston University School of Medicine, Boston, MA USA

**Keywords:** Knee arthroscopy, Complications, Pyogenic arthritis, Venous thromboembolism, Epidemiology

## Abstract

**Background:**

Knee arthroscopy is one of the most common surgical procedures worldwide and the number of arthroscopies has substantially increased in the last 30 years. Thus, our aim was to provide updated estimates on the risk of complications and compare it with the background risk in the general population.

**Methods:**

We identified patients aged 15–84 years with knee arthroscopy in the years 2005–2016 in southern Sweden. We calculated the risk of pyogenic arthritis, venous thromboembolism, and other typical complications within 30 days. As a reference cohort we included the regional population in the corresponding age interval. We estimated the relative and absolute risks of complications compared to the reference cohort using logistic regression adjusted for age, sex, and level of education. We also estimated the proportion of complications in the population explained by knee arthroscopy (population attributable fraction).

**Results:**

We identified 18,735 knee arthroscopy patients (mean age 39 years) and 1,171,084 reference subjects (mean age 46 years). The absolute risk of one or more complications was 1.1% after knee arthroscopy and 0.16% in references. The odds ratio of *any* complication after knee arthroscopy vs. the reference cohort was 9.4 (95% confidence interval [CI] 8.1, 10.9) with an absolute risk difference of 1.4% (1.1, 1.6%). The relative risk (95% CI) for pyogenic arthritis was 115 (75, 174), venous thromboembolism 6.8 (5.1, 9.1), and other complications 7.7 (6.3, 9.5). The population attributable fraction for pyogenic arthritis was 5%.

**Conclusions:**

The absolute risks of complications associated with knee arthroscopy remain small at about 1%. Still, 5% of all pyogenic knee arthritis cases in adults are attributable to knee arthroscopy, thus risks with knee arthroscopy should be carefully considered in the choice of treatment.

## Background

Knee arthroscopy is one of the most common surgical procedures worldwide [1] and the number of arthroscopies has substantially increased in the last 30 years [2]. The patients are often younger or middle-aged physically active individuals. Annually, there are about one million such procedures performed in the United States and in Sweden (population 9.5 million) the corresponding number is about 35,000 [2, 3]. The most common procedures are meniscectomy, meniscal repair, and cruciate ligament reconstruction [2, 4, 5].

Knee arthroscopy is widely acknowledged to be a safe procedure [4, 6]. Still, there are also known serious complications such as joint infection, deep vein thrombosis, pulmonary embolism, and there are even deaths reported [5, 7, 8]. For more complex arthroscopic procedures that involve ligament reconstruction, the risk of complication has been reported to be the highest followed by meniscal repair, chondroplasty, and meniscectomy [5, 7]. However, there is a substantial variation in the reported absolute risks for complications – pyogenic arthritis between 0.08% [4] and 0.42% [9], deep vein thrombosis between 0.12% [5] and 41% [10], and pulmonary embolism between 0.03% [11] and 0.11% [7].

Because knee arthroscopy has become high volume surgery, and the procedure is also more and more frequently performed in older patients, it is of importance to gain updated information on safety of the procedure. Further, there are as of yet *no* studies comparing the risks of complications to the underlying risks for these events in the background population. Such comparisons are needed to get a better understanding of the share of complications attributable to the arthroscopy. Hence, the two main purposes of our study were to: 1) determine updated risks for a number of pre-specified complications within 30 days after knee arthroscopy in absolute terms as well in relative terms compared to the background population, and 2) to estimate the proportion of the total number of complications in the society explained by knee arthroscopies.

## Methods

### Data sources

#### The Skåne Healthcare Register (SHR)

In Skåne region, the most southern part of Sweden, information about all healthcare visits (primary and secondary outpatient care and hospitalizations) is collected in the Skåne Healthcare Register (SHR). Doctors register the diagnostic codes according to the International Classification of Diseases (ICD)-10 system. Codes for surgical procedures are registered according to the classification of healthcare procedures [12]. In addition, information about the type and level of the health care and the date of the visit is included.

#### The Swedish population register

The Swedish population register contains information about deaths, gender, and residential address for all residents. This register is continuously updated by the Swedish Tax Agency.

### Study cohorts and exposure

For both patients having had knee arthroscopy and the reference population we used the same principal inclusion criteria: we required all persons to be *i*) aged 15 to 84 years and *ii*) to be residents in the Skåne region at the time of the index visit.

In our *exposed* cohort, the exposure of interest was a knee arthroscopy (index visit), defined by the surgical codes in the SHR between February 1st 2005 and November 30th 2016. In brief, the selected surgical knee procedures included exploration of the joint, resection of the meniscus, meniscus repair, ligament reconstruction, and synovectomy (Table 1). We included each person only *once*, at the time of their *first* knee arthroscopy during the inclusion period.

**Table 1:** The different knee arthroscopic procedures included in the study

Code^a^	Knee arthroscopic procedure
NGA01	Exploration of soft tissue of knee or lower leg; arthroscopic
NGA11	Exploration of knee joint; arthroscopic
NGA21	Biopsy of soft tissue of knee or lower leg; arthroscopic
NGA31	Biopsy of bone of knee or lower leg; arthroscopic
NGD01	Total excision of meniscus; arthroscopic
NGD11	Partial excision of meniscus; arthroscopic
NGD21	Reinsertion of meniscus; arthroscopic
NGD91	Other operation on meniscus; arthroscopic
NGE01	Incision or suture of joint capsule of knee; arthroscopic
NGE11	Transcision or excision of ligament of knee; arthroscopic
NGE21	Suture or replantation of ligament of knee; arthroscopic
NGE31	Transposition of ligament of knee; arthroscopic
NGE41	Reconstruction of ligament of knee without foreign object; arthroscopic
NGE51	Reconstruction of ligament of knee with foreign object; arthroscopic
NGE91	Other surgery on capsule or ligament of knee; arthroscopic
NGF01	Total synovectomy of knee; arthroscopic
NGF11	Partial synovectomy of knee; arthroscopic
NGF21	Fixation of fragment of surface of knee; arthroscopic
NGF31	Partial excision of joint cartilage of knee; arthroscopic
NGF91	Other operation on synovia or joint surface of knee; arthroscopic
NGH31	Freeing of adhesions of knee joint; arthroscopic
NGH51	Excision of intraarticular exostosis or osteophyte of knee; arthroscopic
NGH71	Surgery for habitual dislocation of knee joint; arthroscopic
NGH91	Other operation of knee joint; arthroscopic

^a^Surgical codes according to “Klassifikation av. vårdåtgärder” (KVÅ) [in Swedish]

**Fig. 1:**
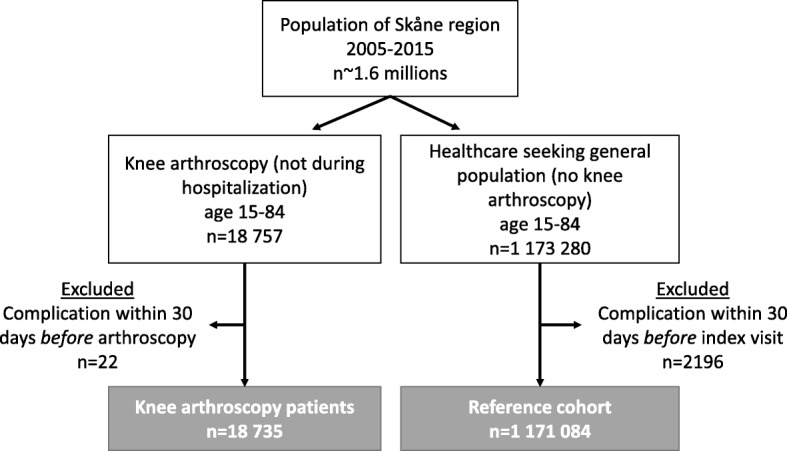
Flow chart of the study cohorts

In our *unexposed* cohort, i.e., background population *not* having knee arthroscopy, we included all persons in the Skåne region with at least one healthcare visit in outpatient care or day surgery (excl. Cases of knee arthroscopy) between the February 1st 2005 and November 30th 2016. We randomly selected an index date from a calendar year with at least one healthcare visit (Fig. 1).

We did not include hospitalizations (i.e. stays at hospital overnight) because all the diagnostic codes are registered once, at the time of discharge. Thus, the true temporal order of diagnoses is unknown, e.g., if pyogenic arthritis developed due to other reasons and was the *indication* to perform the arthroscopic lavage there would have been a risk for reverse causality and thus inflated estimates of risk. Of all knee arthroscopies 6.5% were made during hospitalisations and were thus *not* included in the present study.

### Outcome

The outcome was having at least *one* of our pre-specified complications diagnosed in specialist care within 30 days *afte*r the index visit (Table 2).

**Table 2:** ICD-10 codes for the complications included in the study

ICD-10 code	Complication (Adverse event, outcome). Specialist in and out-patient care	Complication group
I80	Phlebitis and thrombophlebitis	VTE
I26	Pulmonary embolism	
M00^a^	Pyogenic arthritis	Pyogenic arthritis
A41	Other sepsis	Other
L08	Other local infections of skin and subcutaneous tissue	
T81	Complications of procedures, not elsewhere classified	
T81.0	Bleeding and hematoma as complication due to surgical and medical procedures, not elsewhere classified	
T81.1	Post procedural shock	
T81.2	Accidental puncture or damage during surgical and medical procedure, not elsewhere classified	
T81.3	Disruption of wound, not elsewhere classified	
T81.4	Infection following a procedure	
T81.5	Complications of foreign body accidentally left in body following procedure	
T81.6	Acute reaction to foreign substance accidentally left during a procedure	
T81.7	Vascular complications following a procedure, not elsewhere classified	
T81.8	Other complications of procedures, not elsewhere classified	
T81.9	Unspecified complication of procedure	
Y60	Unintentional cut, puncture, perforation or haemorrhage during surgical and medical care	
Y61	Foreign object accidentally left in body during surgical and medical care	
Y69	Unspecified misadventure during surgical and medical care	
Y79	Orthopaedic devices associated with adverse incidents	
Death^b^		

^a^M00 in joints other than the knee were not included

^b^Data on death date were retrieved from the Population Register

The main diagnoses of interest were pyogenic arthritis (ICD-10 code M00), venous thrombotic event (VTE, including thrombophlebitis, ICD-10 code I80 and pulmonary embolism, ICD-10 code I26), complications of procedures (ICD-10 code T81), local infections (ICD-10 code L08), sepsis (ICD-10 code A41) and mistakes done during the surgery (ICD-10 codes Y60, 61, 69 and 71). Primarily, the outcome was having *any* of the aforementioned complications. Additionally, we performed analyses with the specific complication of pyogenic arthritis, VTE, and other complications, respectively.

### Data analysis strategy

To avoid the risk of bias due to reverse causality we only included complications registered from day 1 to day 30 *after* the index visit. The number of complications registered on day 0 was only 15 in the arthroscopy patients and 84 in the reference cohort. In addition, we *excluded* all subjects (both in the arthroscopy group and the reference group) who had *any* of the aforementioned complications up to 30 days *before* the index visit.

We also evaluated the risk for complications after cruciate ligament reconstructions (code NGE41 group within the KVÅ system, Table 1) as compared to the other arthroscopies. Additionally, we estimated the risk of complications after arthroscopy compared to the background population, after excluding cruciate ligament reconstruction from the arthroscopy group. The rationale with these two latter analyses was that cruciate ligament reconstructions have been reported to have a higher risk for complications due to e.g. the drilling of bone tunnels and the typically long duration of the procedure [5, 7].

For the purpose of a sensitivity analysis we created an alternative reference group only containing patients who had consulted for a knee problem but having *no* knee arthroscopy. We used the same study period and age criterion as detailed above. However, we sampled reference subjects, who all have had at least one clinic visit to a physician, with at least one primary diagnostic code for a knee problem which may have represented an indication for knee arthroscopy, but the patient did *not* have knee surgery. The included diagnostic codes were: Meniscus tear, old (M23.2), Chronic instability of knee (M23.5), Unilateral primary osteoarthritis of knee (M17.1), Traumatic tear of meniscus (S83.2), Sprain and strain involving cruciate ligament of knee (S83.5), Other post-traumatic gonarthrosis (M17.3), Tear of articular cartilage of knee, current (S83.3), Other secondary gonarthrosis (M17.5), Injury to multiple structures of knee (S83.7), Patellofemoral disorders (M22.2), Dislocation of patella (S83.0), Other internal derangements of knee (M23.8), and Recurrent dislocation of patella (M22.0). In this sensitivity analysis we also only included arthroscopies with one the above diagnoses registered as main diagnosis. In the analysis, the date of the healthcare visit (reference subjects) or arthroscopy was the index visit. A reference subject was excluded if he/she had any knee surgery (KVÅ code starting with “NG”) within 30 days *before* the index visit as well as a knee surgery other than knee arthroscopy 0 to 30 days *after* the index visit.

### Statistical analysis

We present descriptive data for the study sample as means and standard deviations (SD) for the continuous variables and as counts and percentages for categorical variables. We estimated the odds ratios (ORs) and risk differences using a logistic regression model. We present both crude results (i.e. from an unadjusted model) and results from a model that was adjusted for 3 pre-specified confounders, i.e., characteristics that we considered to be associated with both knee surgery and the risk of complications [13, 14]. These were age (included as continuous variable), sex (male or female), and level of education with four categories (primary school, high school, higher education < 3 years, and higher education ≥3 years). *Individual-level* data on level of education were retrieved from Statistics Sweden (provider of official statistics in Sweden) by register cross-linking. Please note, due to low prevalence of the outcome the ORs can be interpreted as risk ratios [15]. The risk difference was calculated from the adjusted logistic regression model as the difference of the mean probabilities predicted by the model, as implemented in the command *adjrr* in Stata [16]. The population attributable fraction was estimated from the adjusted risk ratios and the prevalence of exposure among cases (i.e. those with outcome) as prevalence_among_cases*((risk_ratio-1)/risk_ratio). All estimates are presented with its 95% confidence intervals (95% CI).

## Results

### Demographics

Out of our 1,171,084 included subjects, 18,735 (1.6%) had undergone knee arthroscopy. Mean age for the knee arthroscopy cohort and the reference cohort was 39.4 years (SD 15.4) and 45.5 years (SD 20.3), respectively (Table 3).

**Table 3:** The description of the study cohorts

	Main analysis		Sensitivity analysis	
	Arthroscopy patients, *n* = 18,735	References (general population), *n* = 1,171,084	Arthroscopy patients, *n* = 15,528	References (knee patients), *n* = 44,804
Age, *mean (SD) years*	39.4 (15.4)	45.5 (20.3)	40.1 (15.4)	49.8 (19.5)
Women, *n (%)*	7398 (39.5)	604,583 (51.6)	5900 (38)	22,721 (51)
Level of education (years), *n (%)*				
Primary school (up to 9)	3824 (21)	282,043 (27)	3114 (21)	11,611 (27)
High school (10–12)	8984 (50)	436,465 (41)	7555 (50)	18,863 (44)
Higher education (13–14)	2419 (13)	141,418 (13)	2057 (14)	5277 (12)
Higher education (15+)	2855 (16)	204,085 (19)	2407 (16)	6803 (16)

### Indications for knee arthroscopy and type of procedures

The four most common diagnoses registered as the primary diagnosis for the knee arthroscopy were: meniscus tear, old (ICD-10 M23.2, 35%), chronic instability of knee (M23.5, 19%), unilateral primary osteoarthritis of knee (M17.1, 11%) and traumatic tear of meniscus (S83.2, 6%).

The three most frequent arthroscopic procedures were partial excision of meniscus (44%), reconstruction of ligament of knee with/without foreign object (17%) and exploration of knee joint (diagnostic arthroscopy) (16%).

### Risk of complications

We found that the risk for any of our pre-specified complications in the knee arthroscopy cohort was 1.1% (210 persons) and in the reference cohort 0.16% (1818 persons) during the follow-up period of 30 days. The most common complications were: complications of procedures not classified elsewhere (33% of all complications), thrombophlebitis (24%) and pulmonary embolism (14%).

The crude OR of having any complication after a knee arthroscopy compared to the reference cohort was 7.3 while it was 9.4 (95% CI 8.1, 10.9) adjusted for age, gender, and level of education (Table 4). The corresponding risk difference was 1.4%. Among the adjusted ORs for the specific complications, pyogenic arthritis yielded by far the highest OR; 115 (95% CI 75, 174). The OR for VTE was 6.8 and for having other complications the OR was 7.7 (Table 4).

**Table 4:** Odds ratios (OR) and risk differences (RD) with 95% confidence intervals (CI) for complications after knee arthroscopy compared to reference persons. Results are from a logistic regression model, crude (unadjusted) or adjusted for age, sex and education level

	Main analysis^a^			Sensitivity analysis^b^		
	Crude	Adjusted		Crude	Adjusted	
	OR (95%CI)	OR (95%CI)	RD (95%CI), %	OR (95%CI)	OR (95%CI)	RD (95%CI), %
Any complication	7.3 (6.3,8.4)	9.4 (8.1, 10.9)	1.35 (1.14, 1.56)	5.0 (3.9, 6.4)	4.9 (3.8, 6.4)	0.95 (0.76, 1.15)
Pyogenic arthritis	127 (85, 191)	115 (75, 174)	0.37 (0.27, 0.47)	13.8 (7.6, 25.1)	10.3 (5.6, 19.0)	0.30 (0.21, 0.39)
VTE^c^	4.8 (3.6, 6.4)	6.8 (5.1, 9.1)	0.37 (0.25, 0.49)	2.0 (1.4, 3.0)	2.3 (1.5, 3.5)	0.17 (0.07, 1.30)
Other	6.4 (5.2, 7.8)	7.7 (6.3, 9.5)	0.61 (0.47, 0.60)	7.8 (5.3, 11.6)	7.9 (5.2, 11.9)	0.54 (0.40, 0.68)

^a^knee arthroscopy compared to general population

^b^knee arthroscopy compared to persons consulting for knee problems

^c^venous thrombotic event

In our sensitivity analysis (the comparison against knee patients but *without* knee arthroscopy) the OR of any aforementioned complication (knee arthroscopy vs. the references) was 4.9 in the adjusted model with an absolute risk difference of 1.0%. The corresponding ORs for pyogenic arthritis, VTE, and other complications were 10.3, 2.3, and 7.9, respectively (Table 4).

The population attributable fraction was 5.1% for pyogenic arthritis, 0.2% for VTE, and 0.3% for the other complications (i.e., about 5% of all pyogenic knee arthritis cases were attributable to the knee arthroscopy).

The OR for complications after *ligament reconstruction surgery* compared to *other arthroscopies* adjusted for age, sex, and level of education was 4.1 (95% CI 2.9, 5.8). The OR for complications after a knee arthroscopy *excluding* ligament reconstruction compared to the reference population was 6.6 (95% CI 5.5, 8.0) with a corresponding risk difference of 0.92% (95% CI 0.73, 1.10%).

## Discussion

The absolute risk of having a complication such as pyogenic arthritis, VTE, or other surgical complications after knee arthroscopy was 1.1%. The increase in risk of these conditions as compared to the general population was about 9-fold, and compared to other patients with a knee condition (but no knee arthroscopy) about 5-fold.

There is a need for updated estimates on the risks associated with knee arthroscopy as there has been an increase in number of procedures, but the procedure is also more and more frequently performed also in middle-aged patients. Also the surgical routines may have improved which calls for a need of new estimates of risks associated with the procedure. Thus, we used comprehensive Swedish health care data over a 12-year time period to evaluate the risks for the most frequent complications during a 30-day follow-up period.

It has been previously reported that knee arthroscopy is associated with complications such as pyogenic arthritis and VTE [4, 7, 10, 17]. However, the estimated over-all risk of complications varies from 0.27 to 8.2% with the risk being higher after more complex and longer lasting arthroscopies [7]. Reigstad et al. [8] presented an over-all risk of 5%, but pain and swelling were included in this study which represented almost 50% of all complications. In our study, we focused on the more *severe* complications which explain why our estimate is lower. Sherman et al. [6] presented the over-all risk of complications after knee arthroscopy to be about 8%. In the study the authors classified complications into minor and major. Several of the *major* complications were more similar to the ones selected in our study such as infections, cardiovascular, neurological and instrument failure. Further, the relatively high over all risk could also potentially partly be explained by the study being 20 years old and that arthroscopic surgery has developed since with better equipment and better surgical routines at large. In the rest of the existing literature, which is somewhat more recent, the reported risk of complications is reported to be below 5%. In one study from Japan [4] the investigators report a very low over all risk for complications of merely 0.27% in a relatively small cohort. In larger cohort studies the risk is typically reported to be between 0.64 and 4.7% [7, 18, 19], which is more in line with our findings. The follow-up period in most studies has typically been up to some 30 days [5, 7, 9, 18, 19] with the exception of a few studies with 90 days of follow-up [8, 11, 18]. In our study, we chose 30 days of follow-up because the most common complications, such as infections and VTE, attributed to the surgery are likely to be diagnosed within that time frame.

Partial meniscectomy was the most common procedure in our study (44% of all knee arthroscopies) which is in line with existing literature [2, 5, 8, 11]. Although arthroscopic partial meniscectomy carries a smaller risk for complications as compared to more complex surgery, such as ligament reconstruction, the reported overall risk is still not negligible [7]. This is in line with our results, where even after *excluding* the ligament reconstruction from the arthroscopy group, the relative risk of a complication was still almost 7-fold as compared to the general population.

Since knee arthroscopy is the most common orthopaedic procedure this results quite many patients experiencing a complication even though the absolute risk is low. For example, our data suggests that 5% of all cases of pyogenic arthritis are caused by knee arthroscopy. Thus, indications for arthroscopic surgery need to be scrutinized as proper evidence of efficacy above non-surgical management is sometimes lacking and compliance to new guidelines still needs to be verified, e.g. for degenerative meniscal lesions and knee osteoarthritis [20, 21].

Our study is based on a large study population from an entire Swedish region without selection bias. The original data is all prospectively registered in the electronic medical records, i.e., free of any potential recall bias from both the patient and doctor. We estimated not only the absolute risk for complications after knee arthroscopy but also the relative risk compared to the background population. Still, we would like to acknowledge some important limitations: As in all register-based research, there is a risk of misclassification of procedures and diagnostic codes which may lead to biased results. However, the validity of the diagnostic codes in SHR and Swedish health care registers in general has been reported to be high [22–24]. We would like to emphasize that these registers are not just administrative data per se*,* as in many other countries, but diagnoses are actually set by the doctors’ themselves and data is drawn directly from the patients’ electronic medical records. Further, we only included complications diagnosed within specialist care, where the validity of the diagnostic codes (such as pyogenic arthritis or VTE) is expected to be higher than in primary care. We did not include the complications registered on day 0 to minimize the risk of reverse causality (pyogenic arthritis). Thus, the true absolute risks may rather have been underestimated, but is not expected to substantially influence the relative risks or the population attributable fraction. Another important limitation is a potential for bias from unmeasured confounding (e.g. smoking and body mass index) and bias by indication – i.e. the systematic differences between persons undergoing arthroscopic surgery and the underlying population. We aimed to take the latter into account in our sensitivity analysis, where only persons with a potential indication for a knee arthroscopy were included which yielded similar results, except for pyogenic arthritis where we noted a substantial attenuation of the risk estimate. Unfortunately, there is no data on body length and body weight available in the register.

## Conclusions

To conclude, although the absolute risk of complication of 1.1% is small, knee arthroscopy should not be considered a completely benign intervention. In particular as it is high volume surgery at most knee surgery clinics. For example, the procedure may be responsible for 5% of cases of pyogenic arthritis of the knee. Thus, it remains important to communicate risks with the patient, and consider non-surgical treatment options when appropriate.

## Abbreviations

CI: confidence interval
ICD: International Classification of Diseases
OR: odds ratio
SHR: Skåne healthcare register
VTE: venous thrombotic event

## References

[1] Treuting R (2000). Minimally invasive orthopedic surgery: arthroscopy. Ochsner J.

[2] Kim S, Bosque J, Meehan JP, Jamali A, Marder R (2011). Increase in outpatient knee arthroscopy in the United States: a comparison of National Surveys of ambulatory surgery, 1996 and 2006. J Bone Joint Surg Am.

[3] Artroskopi- knä [http://www.internetmedicin.se/page.aspx?id=4299]. Accessed Oct 2016.

[4] Hagino T, Ochiai S, Watanabe Y, Senga S, Wako M, Ando T, Sato E, Haro H (2014). Complications after arthroscopic knee surgery. Arch Orthop Trauma Surg.

[5] Jameson SS, Dowen D, James P, Serrano-Pedraza I, Reed MR, Deehan DJ (2011). The burden of arthroscopy of the knee: a contemporary analysis of data from the English NHS. J Bone Joint Surg Br.

[6] Sherman OH, Fox JM, Snyder SJ, Del Pizzo W, Friedman MJ, Ferkel RD, Lawley MJ (1986). Arthroscopy--“no-problem surgery”. An analysis of complications in two thousand six hundred and forty cases. J Bone Joint Surg Am.

[7] Salzler MJ, Lin A, Miller CD, Herold S, Irrgang JJ, Harner CD (2014). Complications after arthroscopic knee surgery. Am J Sports Med.

[8] Reigstad O, Grimsgaard C (2006). Complications in knee arthroscopy. Knee Surg Sports Traumatol Arthrosc.

[9] Armstrong RW, Bolding F, Joseph R (1992). Septic arthritis following arthroscopy: clinical syndromes and analysis of risk factors. Arthroscopy.

[10] Sun Y, Chen D, Xu Z, Shi D, Dai J, Qin J, Qin J, Jiang Q (2014). Deep venous thrombosis after knee arthroscopy: a systematic review and meta-analysis. Arthroscopy.

[11] Hetsroni I, Lyman S, Do H, Mann G, Marx RG (2011). Symptomatic pulmonary embolism after outpatient arthroscopic procedures of the knee: the incidence and risk factors in 418,323 arthroscopies. J Bone Joint Surg Br.

[12] Åtgärdskoder (KVÅ) [http://www.socialstyrelsen.se/klassificeringochkoder/atgardskoderkva]. Accessed Oct 2016.

[13] Wetterholm M, Turkiewicz A, Stigmar K, Hubertsson J, Englund M (2016). The rate of joint replacement in osteoarthritis depends on the patient's socioeconomic status. Acta Orthop.

[14] Rahman MM, Kopec JA, Sayre EC, Greidanus NV, Aghajanian J, Anis AH, Cibere J, Jordan JM, Badley EM (2011). Effect of sociodemographic factors on surgical consultations and hip or knee replacements among patients with osteoarthritis in British Columbia, Canada. J Rheumatol.

[15] Modern Epidemiology, 3rd edn. Edited by Rothman KJ, Greenland S, Lash TL. Philadelphia: Lippincott Williams and Wilkins; 2008. p. 60–1.

[16] Norton EC, Arbor A, Miller MM, Kleinman LC (2013). Computing adjusted risk ratios and risk differences in Stata. Stata J.

[17] Allum R (2002). Complications of arthroscopy of the knee. J Bone Joint Surg Br.

[18] Bohensky MA, deSteiger R, Kondogiannis C, Sundararajan V, Andrianopoulos N, Bucknill A, McColl G, Brand CA (2013). Adverse outcomes associated with elective knee arthroscopy: a population-based cohort study. Arthroscopy.

[19] Martin CT, Pugely AJ, Gao Y, Wolf BR (2013). Risk factors for thirty-day morbidity and mortality following knee arthroscopy: a review of 12,271 patients from the national surgical quality improvement program database. J Bone Joint Surg Am.

[20] Siemieniuk RAC, Harris IA, Agoritsas T, Poolman RW, Brignardello-Petersen R, Van de Velde S, Buchbinder R, Englund M, Lytvyn L, Quinlan C (2017). Arthroscopic surgery for degenerative knee arthritis and meniscal tears: a clinical practice guideline. BMJ.

[21] Beaufils P, Becker R, Kopf S, Englund M, Verdonk R, Ollivier M, Seil R (2017). Surgical management of degenerative meniscus lesions: the 2016 ESSKA meniscus consensus. Knee Surg Sports Traumatol Arthrosc.

[22] Turkiewicz A, Petersson IF, Bjork J, Hawker G, Dahlberg LE, Lohmander LS, Englund M (2014). Current and future impact of osteoarthritis on health care: a population-based study with projections to year 2032. Osteoarthr Cartil.

[23] Rosengren BE, Karlsson M, Petersson I, Englund M (2015). The 21st-century landscape of adult fractures: cohort study of a complete adult regional population. J Bone Miner Res Off J Am Soc Bone Miner Res.

[24] Ludvigsson JF, Andersson E, Ekbom A, Feychting M, Kim JL, Reuterwall C, Heurgren M, Olausson PO (2011). External review and validation of the Swedish national inpatient register. BMC Public Health.

